# Traditional Fermented Foods and Their Physicochemical, Sensory, Flavor, and Microbial Characteristics

**DOI:** 10.3390/foods14203559

**Published:** 2025-10-19

**Authors:** Anjun Chen

**Affiliations:** 1 College of Food Science, Sichuan Agricultural University, Ya’an 625014, China; chen_anjun@sicau.edu.cn; Tel.: +86-0835-2882187; 2 Key Laboratory of Agricultural Product Processing, Nutrition Health (Co-Construction by Ministry and Province), Ministry of Agriculture and Rural Affairs, Ya’an 625014, China

## 1. Introduction

Traditional fermented foods represent a vital cornerstone of culinary cultures worldwide, with a history that spans millennia. Rooted in distinctive production techniques and cultural practices, these foods not only extend the shelf life of perishable ingredients but also generate unique flavors, nutritional benefits, and diverse bioactive properties. However, despite rapid scientific and technological progress, systematic research, knowledge dissemination, and cross-cultural exchange regarding traditional fermented foods remain limited. This gap constrains both a deeper understanding of their complex biological and cultural foundations and the realization of their potential to meet modern flavor demands, drive the green transformation of the food industry, and contribute to sustainable development. Re-examining and systematically investigating traditional fermented foods is therefore not merely a matter of scientific curiosity, but a collective responsibility for preserving dietary diversity, fostering innovation in food systems, and enhancing human well-being.

Against this backdrop, this Special Issue, “Traditional Fermented Foods and Their Physicochemical, Sensory, Flavor, and Microbial Characteristics”, outlines a coherent research trajectory, from sensory and physicochemical characteristics and flavor development, to microbial community succession and molecular mechanisms, microbial interaction dynamics, targeted strain screening, interaction and application, and safety control, culminating in process innovation and the exploration of health benefits. In addition, it also highlights precision fermentation as a source of innovation for alternative proteins, fermented foods, and applications of microorganisms and microbial bioproducts in the food industry. This logical progression provides not only a comprehensive snapshot of the current research frontiers but also key signposts for future academic inquiry and industrial development.

## 2. Contributions to This Special Issue

This Special Issue comprises 12 research articles that collectively advance our understanding of traditional fermented foods from multiple perspectives.

Flavor and sensory perception represent decisive factors influencing consumer acceptance of fermented foods. In Mediterranean countries, artisan bread has gained remarkable popularity, especially following the COVID-19 pandemic, due to its reliance on simple recipes, natural ingredients, and traditional craftsmanship. Sanmartín and co-workers [1] investigated 16 artisan breads from the Valencia region of Spain, examining their volatile profiles and sensory attributes. The study demonstrated that bread dough pH, total titratable acidity, and acid content were influenced by flour type and sourdough use. Whole wheat and high-ash flours further promoted acetic acid production, enriching flavor complexity. Artisan breads contained 51–55 volatile organic compounds (VOCs), far exceeding the <20 VOCs detected in industrial breads, with aldehydes, alcohols, acids, and furans being the primary components. Flour type determines specific volatiles; for example, spelt flour correlated with 2-methylbutanol, durum wheat with 3-methylbutanol, and T80 flour with 3-decen-1-ol and 5-decen-1-ol. Sensory evaluation confirmed that raw materials and starter cultures directly shaped flavor intensity, acidity, color, and texture. Importantly, this work provides quantitative guidance for artisanal bakers and advancing differentiated strategies for the baking industry.

As a representative of traditional Chinese fermented vegetables, Sichuan paocai embodies both profound local culinary heritage and substantial relevance to the modern food industry. However, with the advancement of industrial production, the widespread use of high-salt pickling techniques, while ensuring product safety and stability, has also led to flavor homogenization and reduced microbial diversity, thereby diminishing the unique sensory attributes of traditional paocai. To address this challenge, Xian and co-workers [2] systematically compared the physicochemical characteristics, flavor profiles, and microbial communities of industrially produced Sichuan paocai fermented using traditional techniques across seven regions. Their objective was to elucidate the mechanisms through which regional “terroir” shapes product quality, thereby providing a scientific basis for reproducing traditional flavor diversity within industrial systems. The study revealed significant regional variations in physicochemical indicators such as acidity, salt content, and color. In total, 294 volatile flavor compounds were identified, with alcohols, esters, and isothiocyanates serving as the primary contributors. Notably, samples from Chengdu and Meishan displayed superior aroma intensity and greater chemical diversity, significantly outperforming household-scale products. Microbial community analysis showed that lactic acid bacteria (LAB), including *Lactobacillus*, *Pediococcus*, and *Weissella*, dominated the fermentation process, with marked regional differences in their relative abundance and diversity. LEfSe analysis further identified region-specific microbial biomarkers: Meishan exhibited the highest microbial diversity, while Chengdu was dominated by *Lactobacillus*. Correlation analysis revealed strong positive associations between LAB and key flavor compounds, underscoring their central role in flavor formation. Additionally, isothiocyanates inhibited undesirable microorganisms, thereby contributing to system stability. Overall, this research deepens our understanding of how regional terroir shapes the quality of fermented foods, while also providing critical scientific evidence to support regional branding, process optimization, and geographical indication protection in the Sichuan paocai industry.

The dynamic succession of microbial communities constitutes a central mechanism in fermentation processes. In northwestern Lao PDR, the Luar ethnic group has long produced “Miang”—a chewing tea prepared from steamed Assam tea leaves compressed in bamboo tubes and fermented underground for 3–5 weeks—which serves both as a snack and a cultural symbol. Yet systematic investigations into microbial succession, bioactive compound dynamics, and quality assurance in underground fermentation remain scarce. Phovisay and co-workers [3] tracked Miang fermentation at four stages (0, 1, 3, and 5 weeks). Their results showed that *Lactobacillus* and *Pichia* rapidly became dominant within one week and completely suppressed potential pathogens by week three. Simultaneously, reductions in pH, increases in total acidity, and heightened activities of pectinase, β-mannanase, and cellulase promoted crude fiber degradation and texture softening. Polyphenol content increased markedly (from 103.5 mg/g to 144.9 mg/g), while tannin levels decreased by 22%, yielding a smoother taste. Collectively, these findings establish Miang as a model of traditional fermented tea in which lactic acid bacteria–yeast co-metabolism simultaneously ensures safety, enhances functional value, and improves sensory properties.

Metaproteomics has recently emerged as a powerful tool for elucidating the molecular mechanisms underlying probiotic and bioactive functionalities in fermented vegetables. Rueangsri and co-workers [4] conducted a comparative study of microbial community dynamics and protein expression profiles in fermented vegetables produced under three formulations: standard, probiotic-supplemented, and antioxidant-enriched. Across all samples, *Leuconostocaceae* and *Lactobacillaceae* predominated; however, α-diversity differed significantly depending on treatment and fermentation stage. Probiotic supplementation promoted the enrichment of *Lacticaseibacillus rhamnosus* and *Pediococcus*, while the addition of polyphenols stimulated the proliferation of *Levilactobacillus brevis*, *Weissella*, and other taxa. Multivariate analyses, including clustering and PLS-DA, clearly distinguished microbial and protein expression profiles between day 0 and day 15 samples. Several proteins displayed unique associations with antioxidant activity, suggesting their involvement in oxidative stress during vegetable fermentation. Moreover, a longitudinal comparison between day 0 and day 15 revealed proteins linked to DNA replication and repair, such as transcription elongation factor GreA, tRNA pseudouridine synthase B, and various helicases. These findings not only enhance our understanding of the functional roles of LAB-derived proteins in vegetable fermentation but also provide molecular evidence for improving texture, flavor, and health-promoting properties of fermented vegetables through targeted microbial regulation.

As consumer demand for natural and sustainable preservation methods grows, lactic acid bacteria and their metabolites—especially bacteriocins such as enterocins—have attracted wide interest as alternatives to synthetic preservatives. Algerian traditional dried figs marinated in olive oil are nutrient-rich but underexplored as a source of probiotic and antimicrobial strains. To investigate this potential, Merzoug and co-workers [5] isolated and identified 12 *Enterococcus faecium* strains. Species confirmation was performed via MALDI-TOF MS and 16S rRNA sequencing, genetic diversity was assessed using BOX-PCR, GTG-PCR, and ERIC-PCR, antibacterial activity was evaluated against five foodborne and clinical pathogens by well diffusion, and protease sensitivity tests confirmed the proteinaceous nature of active substances. Additionally, PCR screening of 11 enterocin genes, 3 virulence genes, and 5 antibiotic resistance genes was performed for safety assessment. Results showed that all 12 isolates were *E*. *faecium*, with substantial genetic diversity. Nine strains displayed strong antibacterial activity, with strain HFM7 (carries *entA*, *entL50A*, and *entL50B*) exhibiting the largest inhibition zone (20.0 ± 1.0 mm). All antimicrobial activities were lost upon protease treatment, confirming the active compounds as bacteriocins. Importantly, no virulence or antibiotic resistance genes were detected, supporting their safety for food applications. This study highlights Algerian dried figs in olive oil as an untapped reservoir of safe and functional *E*. *faecium* strains, providing a valuable resource for the development of “clean-label” natural preservatives and starter cultures.

Interactions between dominant microbial populations critically shape flavor development in fermentation. Sichuan Baoning bran vinegar, produced via solid-state fermentation with uncooked wheat bran as the primary substrate, is characterized by a distinctive “high lactic acid and low acetic acid” profile. However, the interplay between the dominant lactic acid bacterium *Lactobacillus amylovorus* and the acetic acid bacterium *Acetobacter pasteurianus* during vinegar fermentation has not been systematically examined. To address this, Li and co-workers [6] isolated *L*. *amylovorus* LL34 (a high lactic acid producer) and *A*. *pasteurianus* LA10 (a high acetic acid producer) from Baoning vinegar and performed pure-culture and co-culture experiments in a simulated fermentation system. Results showed that LA10 strongly inhibited LL34 growth, while LL34 partially suppressed the acid production of LA10, resulting in lower total and acetic acid contents in co-culture compared with LA10 alone. At the volatile level, LA10 pure culture generated the highest number of compounds (39), followed by the co-culture (36) and LL34 (34). Additionally, metabolomic analysis revealed 771 differential non-volatile metabolites, with the co-culture significantly enriching functional compounds such as phenyllactic acid, 3-phenyllactic acid, 4-vinylphenol, and 6-hydroxyhexanoic acid. The enrichment of acid and amino acid metabolic pathways was also observed. Collectively, these findings provide mechanistic insights into how microbial interactions modulate the acid balance and aroma complexity of Sichuan bran vinegar, thereby contributing to its unique sensory identity.

As research on fermentation mechanisms continues to advance, increasing attention has been directed toward the application potential of specific functional strains. Within the expanding specialty coffee market, consumers demand both unique flavors and health attributes. Traditional spontaneous fermentation is frequently associated with contamination risks and batch inconsistency, whereas directed yeast inoculation offers greater control and quality improvement. While *Kluyveromyces lactis* is well recognized for its aromatic compound production in cheese and wine fermentation, its role in coffee fermentation has remained unexplored. Abreu and co-workers [7] conducted a comparative investigation of *K*. *lactis* and the conventional fermentation yeast *Saccharomyces cerevisiae* during the wet fermentation of four Arabica coffee cultivars. Their results demonstrated that *S*. *cerevisiae* CA11 promoted the higher retention of trigonelline and chlorogenic acids in MGS Paraíso 2 (P2) and Catuai Amarelo IAC62 (CA62), while *K*. *lactis* B10 fermentation elevated chlorogenic acid levels only in P2 and MGS Catucaí Pioneira (CP). Furthermore, *S*. *cerevisiae* CA11 imparted fruity and floral notes, particularly in CP and P2 varieties, whereas *K*. *lactis* B10 was associated with sensory attributes such as lemongrass, citrus, and honey-lemon nuances. Importantly, the use of a starter culture enabled the classification of coffees as specialty grade, underscoring the potential of directed microbial inoculation in elevating coffee quality and market value.

Traditional Chinese fermented fish products (CTFPs) are highly valued for their distinctive flavor but face critical safety concerns, as more than 60% of commercially available products contain N-nitrosamines (NAs) at levels exceeding safety thresholds, posing carcinogenic risks. The main precursors of NAs are biogenic amines (e.g., putrescine, cadaverine, tyramine) generated by protein degradation, and nitrites commonly introduced during processing. These compounds readily react in the acidic gastric environment, leading to NAs formation. To mitigate this risk, Li and co-workers [8] evaluated the potential of *Lactobacillus plantarum* 120 (*Lactiplantibacillus*), *Saccharomyces cerevisiae* 2018, and *Staphylococcus xylosus* 135, both individually and in mixed cultures, as starter agents. Using in vitro simulations of the entire digestive tract, they systematically examined the dynamic changes in microbial populations, biogenic amines, nitrites, and NA levels during the digestion of CTFPs. Results indicated that gastric acidity markedly inhibited microbial survival and reduced nitrite levels, but simultaneously enhanced nitrosation reactions, thereby increasing NA levels—particularly of N-nitrosodimethylamine and N-nitrosopiperidine. Upon entry into the small intestine, microbial growth resumed, accompanied by an increased accumulation of biogenic amines (except spermidine and spermine) and nitrites. Compared with spontaneous fermentation, the inoculated group significantly reduced N-nitrosodimethylamine formation during gastric digestion and suppressed the accumulation of biogenic amines and nitrites in the intestinal phase. These findings provide a feasible strategy to improve the digestive safety of fermented fish products through the use of locally sourced composite starter cultures.

The Sichuan paocai industry is one of the largest sectors of Chinese fermented foods, yet the disposal of “high-salt brine” byproducts remains a critical bottleneck to sustainable development. Conventional two-stage fermentation employs low-salt brine (<5%) to initiate flavor formation, followed by high-salt brine (>10%) for preservation, leading to the accumulation of waste brine rich in organic acids that are difficult to recycle. To address this, Xian and co-workers [9] performed a systematic comparison of two pre-dehydration methods—hot-air drying (HAP) and salt-pressure dehydration (SP)—with fresh vegetable direct fermentation (SFP) as a control. Radish strips were dehydrated to 55–60% of their original weight and fermented at 20 °C in 7% brine with aged brine supplementation. Using high-throughput sequencing, the authors tracked the dynamics of bacterial and fungal communities while simultaneously measuring 17 free amino acids, 6 organic acids, and key physicochemical parameters (pH, titratable acidity, salinity, color, and texture). Results indicated that although pre-dehydration delayed fermentation speed, it significantly increased titratable acidity and umami amino acid levels (lysine and glutamic acid with TAV > 1), producing a richer flavor profile. Importantly, hot-air drying reduced the initial salinity to levels comparable to traditional high-salt fermentation, thereby substantially lowering brine requirements. Core microbial taxa—including *Lactobacillus*, *Weissella*, *Enterobacter*, *Wallemia*, *Aspergillus*, and *Kazachstania*—dominated all treatments, but dehydration reshaped community structures by reducing network complexity, enhancing modularity, and improving stability. Moreover, LEfSe analysis identified 23 bacterial and 43 fungal biomarkers whose correlations with non-volatile compounds varied across treatments but which had limited effects on dominant taxa. Taken together, these findings demonstrate that raw-material pre-dehydration, particularly hot-air drying, can effectively reduce high-salt brine byproducts while maintaining or enhancing paocai quality, offering a promising strategy that reconciles environmental sustainability with industrial profitability.

Within the global pursuit of healthier diets and sustainable agricultural practices, plant-based fermented foods have attracted growing scientific attention owing to their probiotic potential, high dietary fiber, and abundant natural antioxidant potential. Thailand, renowned for its diverse vegetable resources and fermentation practices, provides a representative case. Pan-utai and co-workers [10] systematically analyzed 45 commercial samples of ten representative fermented vegetables from six regions of Thailand. Their findings revealed that fermented tea leaves and pickled bamboo shoots exhibited the highest total phenolic contents. Notably, pickled mustard greens demonstrated pronounced antioxidant capacity, with DPPH (1,1-diphenyl-2-picrylhydrazyl radical) and ABTS (2,2′-azino-bis (3-ethylbenzothiazoline-6-sulfonic acid) diammonium salt) inhibition rates of 83.77% and 94.26%, respectively. Multivariate analysis further clustered the vegetables into four groups with distinct chemical profiles, in which lipids, titratable acidity, protein, fiber, salinity, and total soluble solids served as key discriminating factors. These results underscore the finding that both raw material selection and processing strategies shape the nutritional, physicochemical, and antioxidant characteristics of fermented vegetables, thereby offering promising resources for dietary health promotion and industrial innovation.

The high-value application of traditional fermented foods remains a key direction in fermentation science. Among these, traditional vinegars have garnered attention for their potential antioxidant, anti-aging, and skin-brightening properties. Yu and co-workers [11] systematically evaluated 15 Korean grain vinegars and 14 persimmon vinegars, with distilled white vinegar as a control, to assess their potential for anti-wrinkle and whitening functions. Physicochemical parameters, antioxidant activities, and collagenase and tyrosinase inhibition assays were conducted, and Pearson correlation analysis was applied to examine associations between composition and function. The results revealed that grain vinegars exhibited collagenase inhibition ranging from 3.57% to 100% and tyrosinase inhibition from 62.38% to 77.03%, whereas persimmon vinegars ranged from 0% to 94.5% and 30.75% to 71.54%, respectively. The functional activity of grain vinegar correlated positively with total nitrogen, free amino acids, and antioxidant capacity, while persimmon vinegar was positively associated with organic acids such as lactic acid. In both cases, higher residual sugar content was inversely correlated with functional activity, underscoring the importance of controlling fermentation endpoints. These findings provide scientific evidence for positioning traditional Korean vinegars as high-value ingredients in cosmetics and health foods, thus broadening their application beyond the food sector.

Against the backdrop of a continuously expanding global population and intensifying climate change, traditional food production systems are confronted with multiple challenges, including resource scarcity, environmental pressures, and food safety risks. Within this context, microbial technology, characterized by its green, efficient, and sustainable attributes, has emerged as a pivotal driver of transformation in the food industry. Alane Beatriz Vermelho and co-workers [12] conducted a systematic review of recent advances in microbial applications for sustainable food production. The review highlights five major domains: microbial bioproducts used in the food sector; the fermentation process and its products; microbial food additives and ingredients such as organic acids, texturizers and stabilizers, colorants, sweeteners, and flavoring and aroma compounds; probiotics, prebiotics, and postbiotics; and methodologies for evaluating contamination and food preservation. Research indicates that liquid fermentation, solid-state fermentation, and precision fermentation not only enable the production of functional components such as enzymes, organic acids, and natural pigments, but also facilitate the utilization of agricultural byproducts as substrates, thereby promoting resource recycling and advancing the circular bioeconomy. Moreover, naturally occurring antimicrobial substances produced by microorganisms—such as bacteriocins, biopolymers, and bacteriophages—show strong potential to replace chemical preservatives and extend food shelf life. In addition, the integration of biosensors and nanotechnology allows microorganisms to contribute to the rapid detection of foodborne pathogens, thereby enhancing food safety standards. Despite these opportunities, significant challenges remain, including difficulties in large-scale production, inadequate cost control, and complex regulatory frameworks. Looking forward, advances in synthetic biology, multi-omics technologies, and intelligent biomanufacturing are expected to empower microbial technology to play an increasingly critical role in shaping a safe, nutritious, and environmentally sustainable future food system.

## 3. Conclusions

As Guest Editor, I sincerely hope that this Special Issue will serve as a valuable resource for scholars, practitioners, and industry stakeholders, inspiring new approaches to both scientific inquiry and technological innovation in the field of fermentation. We extend our deepest gratitude to all contributing authors for their commitment, creativity, and scholarly rigor.

## Data Availability

Not applicable.
